# Protein Engineering of a Pyridoxal-5′-Phosphate-Dependent l-Aspartate-α-Decarboxylase from *Tribolium castaneum* for β-Alanine Production

**DOI:** 10.3390/molecules25061280

**Published:** 2020-03-12

**Authors:** Xin-Jun Yu, Chang-Yi Huang, Xiao-Dan Xu, Hong Chen, Miao-Jie Liang, Zhe-Xian Xu, Hui-Xia Xu, Zhao Wang

**Affiliations:** Key Laboratory of Bioorganic Synthesis of Zhejiang Province, College of Biotechnology and Bioengineering, Zhejiang University of Technology, No.18, Chaowang Road, Hangzhou 310014, China; xjyu@zjut.edu.cn (X.-J.Y.); hcy929390419@gmail.com (C.-Y.H.); xuxiaodan7@163.com (X.-D.X.); hzchenhong1016@163.com (H.C.); mjliang2008@163.com (M.-J.L.); xuzhexian1231@126.com (Z.-X.X.); xhx1062@163.com (H.-X.X.)

**Keywords:** β-alanine, pyridoxal-5′-phosphate (PLP), l-aspartate-α-decarboxylase, directed evolution, characterization, whole-cell bioconversion

## Abstract

In the present study, a pyridoxal-5′-phosphate (PLP)-dependent L-aspartate-α-decarboxylase from *Tribolium castaneum* (TcPanD) was selected for protein engineering to efficiently produce β-alanine. A mutant *Tc*PanD-R98H/K305S with a 2.45-fold higher activity than the wide type was selected through error-prone PCR, site-saturation mutagenesis, and 96-well plate screening technologies. The characterization of purified enzyme TcPanD-R98H/K305S showed that the optimal cofactor PLP concentration, temperature, and pH were 0.04% (*m/v*), 50 °C, and 7.0, respectively. The 1mM of Na^+^, Ni^2+^, Co^2+^, K^+^, and Ca^2+^ stimulated the activity of TcPanD-R98H/K305S, while only 5 mM of Ni^2+^ and Na^+^ could increase its activity. The kinetic analysis indicated that TcPanD-R98H/K305S had a higher substrate affinity and enzymatic reaction rate than the wild enzyme. A total of 267 g/L substrate l-aspartic acid was consumed and 170.5 g/L of β-alanine with a molar conversion of 95.5% was obtained under the optimal condition and 5-L reactor fermentation.

## 1. Introduction

The β-alanine (3-aminopropionic acid) is the only β-type amino acid in nature and non-protein amino acid in vivo, which is not involved in protein synthesis, while it has various biological functions. Thus, the β-alanine is widely used in multiple industries, such as the medicine, food, and biochemical industry. In the medicine industry, β-alanine is used to synthesize a series of high-value products, such as pantothenic acid, calcium pantothenate, carnosine, disodium pamidronate, balsalazide, and so on [1,2,3]. In the food industry, β-alanine serves as a food additive to improve the flavour of seasonings. Besides, β-alanine is an important nutritional supplement for athletes to improve their body functions [4,5]. The chemical method is the traditional routine for β-alanine production [6,7,8]. However, high temperature and pressure, and strong acid and base, are used in the chemical routine for β-alanine production, leading to heavy environmental pollution. Moreover, numerous byproducts are produced under harsh condition due to the uncontrollability of the chemical reaction. Thus, the alternative green, mild, and controllable method for β-alanine production must be developed and applied.

The enzymatic method for β-alanine production, which belongs to a biological routine, is more attractive due to its mild reaction condition, high specificity, and less environmental pollution [3,9]. l-aspartate-α-decarboxylase (EC 4.1.1.11, PanD) is a class of enzyme catalyzing the decarboxylation of L-aspartate to produce β-alanine. PanD is grouped into two types based on the active groups, the pyruvate-dependent type and the pyridoxal-5′-phosphate (PLP)-dependent type, respectively. The pyruvate-dependent PanD is only active when pyruvate is present as a cofactor and mainly distributed in prokaryotes, especially in bacteria [10]. The other PanD, the PLP-dependent type, only shows its activity with the PLP as a cofactor and it is mainly found in eukaryotes, such as plants and insects [11]. In recent years, most studies mainly focus on the PanDs from prokaryotes for β-alanine productions, such as *Escherichia coli*. [12], *Corynebacterium glutamicum* [13], *Bacillus subtilis* [14], and *Mycobacterium tuberculosis* [15]. However, pyruvoyl-dependent PanD derived from prokaryotes inactivate under the turnover conditions, irreversibly. The further study shows the inactivation that arises from a side reaction, which was similar to the inactivation mechanism of the S-adenosylmethionine decarboxylase [16,17]. Thus, the inactivity of the pyruvoyl-dependent PanD inhibits its application in β-alanine production. The PLP-dependent type of PanDs are also used for β-alanine production to avoid the spontaneous inactivity of PanD. The PanD genes from *T. castaneum*, *E. coli*, and *C. glutamicum* were expressed in *Saccharomyces cerevisiae* for the *de novo* biosynthesis of β-alanine, suggesting that the TcPanD played an important role in increasing the β-alanine yield [18].

The protein engineering technology, which is based on directed evolution of enzyme, is widely used to improve enzymatic characteristics, such as activity and stability, for β-alanine production. The *PanD* gene that was derived from *B. subtilis* was randomly mutated by error-prone PCR, and the mutants (I88M and V68I) were screened, with 18–22% higher specificity and 29–64% higher stability when compared with the wild-type [19]. Zhang et al. obtained a mutant PanD derived from *Bacillus substilis* by site-directed mutagenesis and expressed in *Escherichia coli* which showed a 1.6-fold higher activity and 1.4-fold increased residual activity than the wild-type [14]. In the present study, the PanD derived from *Tribolium castaneum* was selected for protein engineering in order to improve its enzymatic activity and catalytic stability. The mutant with the highest enzymatic activity was selected, and the characteristic profiles of the mutant and wild enzymes were compared and analyzed, explaining why the mutant enzyme has higher activity and stability than the wild enzyme. The availability of β-alanine production while using the *E.coli* transformant carrying the mutant PanD gene was evaluated at a 5-L level.

## 2. Results and Discussion

### 2.1. Protein Engineering of TcPanD

**Figure 1:**
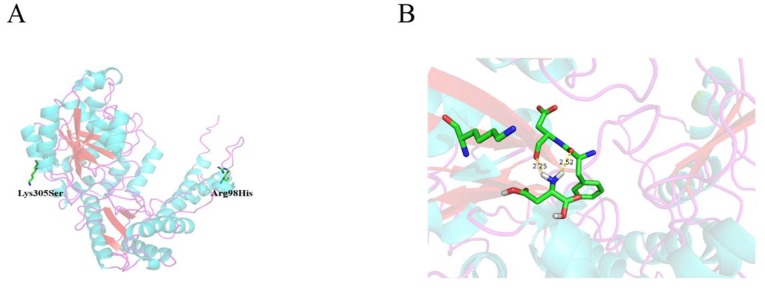
Three-dimensional structure of TcPanD (**A**) and Auto docking with l-Asp (**B**).

The *TcPanD* gene from *T. castaneum* was randomly and then site-directed mutated, and the mutants were cloned into pET-28a(+) and expressed in *E. coli* BL21(DE3). Two libraries comprising 3500 random mutants and 1000 sited-directed mutants were constructed by error-prone PCR with a mutation rate of approximately 0.25%. Two mutants, named 16-H5 and 18-G6, with higher β-alanine yield, were screened using the random mutation technology, and their enzymatic activities were 1.61- and 1.43- times higher than that of wild *TcPanD*. The sequencing analysis showed that the mutation sites were R98H, K305E, and I451V, respectively. Subsequently, these three sites were site-directed saturation mutated, and the mutant *TcPanD-R98H/K305S* with the enzymatic activity 2.45 times higher than that of the wild *TcPanD* was obtained. Figure 1A shows the homology modeling of TcPanD and the varied amino acid sites are located at the random coil of the surface, forming a large amount of irregularly coiled structures, which connect α-helix and β-sheet to form a stable three-dimensional structure. Therefore, even if the varied amino acid sites are located on the edge of irregular curl, they still affect the TcPanD activity. The three-dimensional structure of the mutant TcPanD-R98H/K305S was docked with L-aspartic acid to obtain a putative substrate pocket, as shown in Figure 1B. The residue R98 is distant from substrate L-aspartate, and the relative enzyme activity is 1.89 times higher than that of the wild enzyme TcPanD after a single point mutation. It is speculated that R98 might play an important role in structural stabilization, which has a certain impact on the improvement of catalytic activity. The residue K305 is located near the substrate L-aspartate. The site is mutated from lysine to serine, and serine belongs to a hydroxy amino acid and it has strong hydrophilicity, which is beneficial to the tight binding of the enzyme to the substrate. When the steric hindrance of the electron sink is small, it is more inclined to attack the α-carboxyl, which has a larger steric hindrance and stronger electronegativity. While in a large steric hindrance, the electron sink is easier to attack the small steric hindrance β-carboxyl to form a Schiff base to complete the decarboxylation process. As an electron sink, PLP is conducive for l-aspartate to transform into a carbanion transition structure and, thus, facilitates the decarboxylation [20]. From the analysis of steric hindrance, the residue of serine is shorter than that of lysine, which leads to a smaller steric hindrance, so that the reaction of α-decarboxylation might be easier to happen. This mutation mechanism might also relevant to the improved enzymatic stability of temperature and pH for the mutant TcPanD-R98H/K305S, as discussed below. This mechanism might also explain why the activity of the mutant TcPanD-R98H/K305S is more sensitive than that of the wild-type TcPanD, as discussed below.

### 2.2. Purification and Characterization of the TcPanD-R98H/K305S

**Figure 2:**
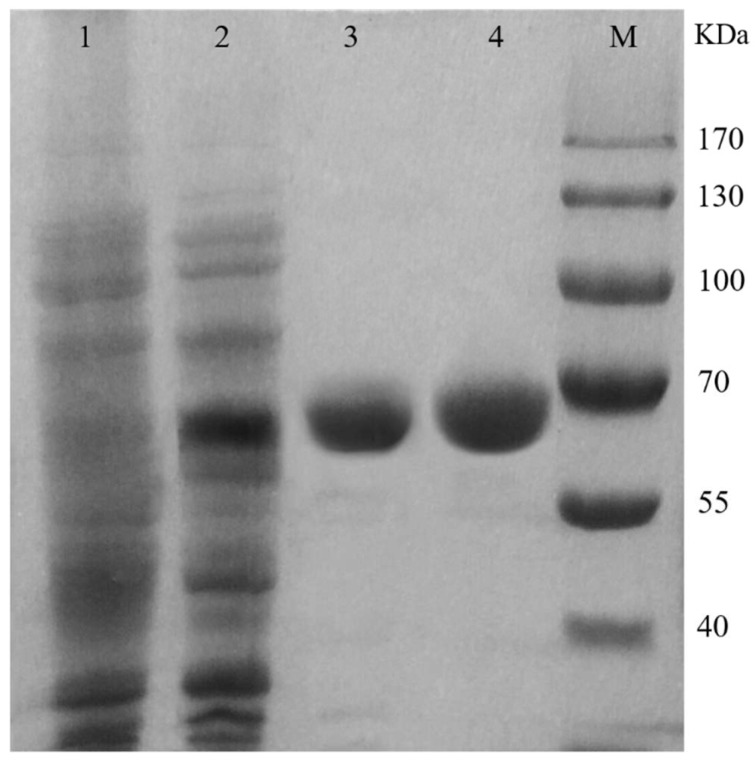
SDS-PAGE electrophoresis map of PanD purification Lane M, Protein Marker; Lane 1, empty control of *E.coli* BL21/pET28; Lane 2, crude enzyme; Lane 3, the sample of purified mutant TcPanD-R98H/K305; Lane 4, the sample of the purified wild enzyme TcPanD (approximate molecular weight 65 kDa).

The mutant TcPanD-R98H/K305S has the highest enzymatic activity and it is purified and characterized for further study. Table 1 shows the summary for the purification steps of the wild TcPanD and the mutant TcPanD-R98H/K305S. The supernatant of TcPanD was filtrated through a 10 kDa membrane with a yield of 44.89%, thereby resulting in a total protein amount of 19.05 mg and a 2.14-fold increase in a specific activity of 2.88 U/mg. The mutant TcPanD-R98H/K305S was purified 2.77-fold from the crude enzyme solution after the 10 kDa membrane ultrafiltration steps, with 41.77% recovery and an observed specific activity of 7.046 U/mg. The specific enzyme activity of the mutant TcPanD-R98H/K305S was 2.45 times higher than the wild TcPanD. The SDS-PAGE analysis of the purified PanD showed a single band (Figure 2), and the molecular weights of TcPanD and TcPanD-R98H/K305S were 65.0 kDa, which was similar to the theoretical value. The molecular mass of the PanD that was derived from eucaryotic organisms was higher than those that were derived from prokaryotic organisms, such as *E. coli*, *B. subtilis*, and *C. glutamicum* [14].

**Figure 3:**
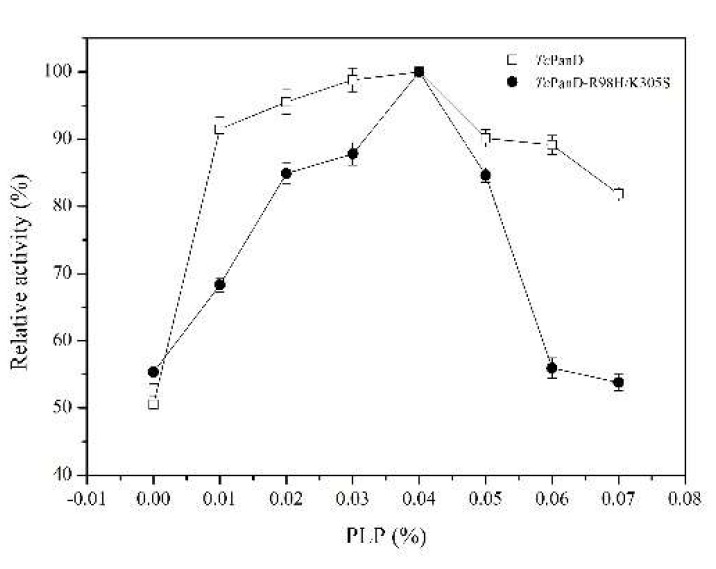
Effect of pyridoxal 5’-phosphate (PLP) addition on TcPanD and TcPanD-R98H/K305S. Data are given as mean ± SD, n = 3.

PLP acts as a cofactor and plays an important role for the catalysis of TcPanD, as the TcPanD belongs to a pyridoxal 5’-phosphate (PLP)-dependent decarboxylase. The results from Figure 3 showed that, with the increase in PLP, the enzymatic activities of the two proteins reached the highest levels under the treatment of 0.04% PLP. In addition, the mutant TcPanD-R98H/K305S was shown to be more sensitive to PLP than the wild TcPanD. Thus, the PLP is a potential regulator in β-alanine production.

**Figure 4:**
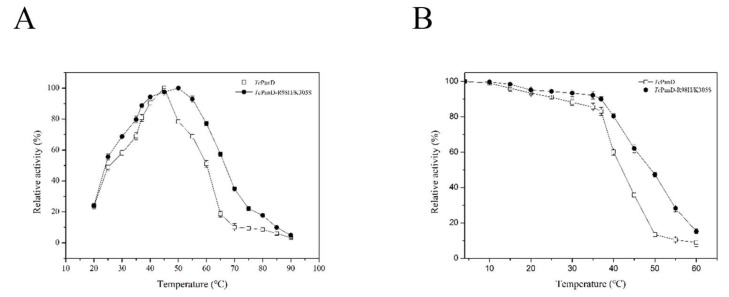
Effect of temperature on TcPanD and TcPanD-R98H/K305S (**A**) and thermostability (**B**). A, The optimal temperature of TcPanD and TcPanD-R98H/K305S; B, The thermal stability of TcPanD and TcPanD-R98H/K305S at different temperatures (4–60 °C). Data are given as mean ± SD, n = 3.

Temperature is an important factor affecting the rate of an enzymatic reaction. The optimum temperature of TcPanD was 45 °C, while that of TcPanD-R98H/K305S was 50 °C, as shown in Figure 4A. Moreover, the TcPanD activity remained more than 90% at a range of 40–45 °C and it was lost rapidly at above 45 °C. The TcPanD-R98H/K305S activity remained more than 90% of the highest activity at a range of 40–55 °C. The residual enzyme activity of the wild TcPanD was relatively stable at a temperature range of 4–37 °C, as shown in Figure 4B. When the temperature reached the optimal level (45 °C), the activity of the wild TcPanD only remained 35.73%. Meanwhile, the mutant TcPanD-R98H/K305S was relatively stable at a temperature range of 4–60 °C. When the temperature reached up to 50 °C, the residual activity of the TcPanD-R98H/K305S remained 47.38%. Thus, the mutant TcPanD-R98H/K305S showed better thermostability than the wild TcPanD. Although the optimal temperatures of TcPanD and TcPanD-R98H/K305S were 45 °C and 50 °C, respectively, the enzyme could remain stable at 37 °C. Thus, the optimal temperature for the enzyme is 37 °C, at which it had the highest activity and thermostability.

**Figure 5:**
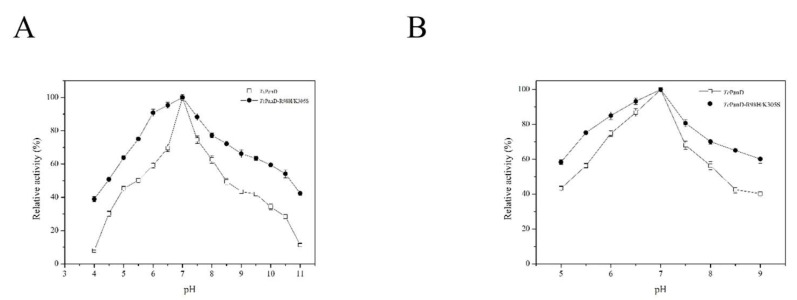
Effect of pH on TcPanD and TcPanD-R98H/K305S (**A**) and pH stability (**B**). A, The optimal pH of TcPanD and TcPanD-R98H/K305S; B, The pH stability of TcPanD and TcPanD-R98H/K305S at different pHs (5.0–9.0). Data are given as mean ± SD, n = 3.

The optimal pH for the mutant TcPanD-R98H/K305S and the wild TcPanD were both 7.0, which was like that from *B. subtilis*., as shown in Figure 5A [14]. As pH was increased from 7.0 to 11.0, the enzymatic activity of TcPanD decreased more sharply than TcPanD-R98H/K305S. The pH stability for the TcPanD and TcPanD-R98H/K305S was also analyzed. The residual activity of TcPanD was stable at a pH range of 6.0–7.5, as shown in Figure 5B. The residual activity of TcPanD-R98H/K305S could remain more than 70% at a pH range of 5.5–8.5. The residual activity of TcPanD-R98H/K305S remained about 62.16% at pH 9.0, while the residual activity of wild TcPanD remained 40.28% at pH 9.0. These results suggested that the mutant TcPanD-R98H/K305S had higher pH stability than the wild TcPanD. High pH stability can reduce the inorganic salt content in the reaction system and facilitate subsequent purification in industrial production.

**Figure 6:**
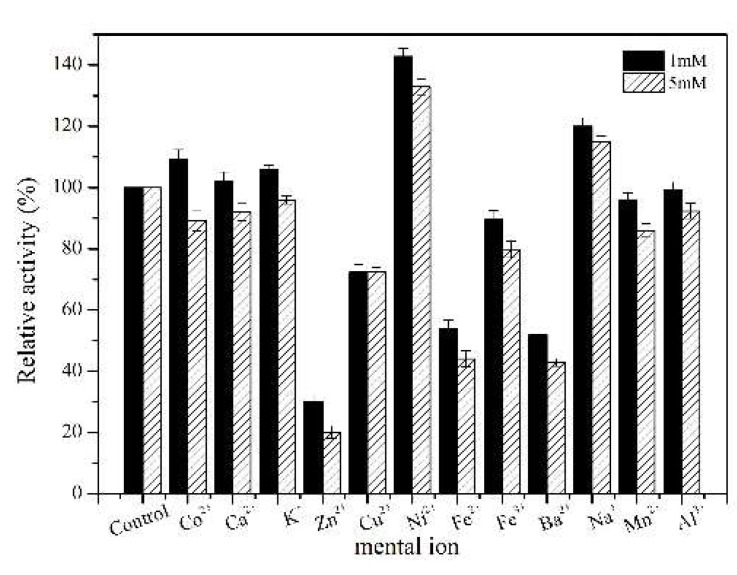
Effects of metal ions on the activity of TcPanD-R98H/K305S.

The metal ion is one of the important factors affecting the activity of the enzyme. In this study, the effects of metal ions on the wild TcPanD and the mutant TcPanD-R98H/K305S activity at two different concentrations (1 and 5 mM) were investigated. Under the low metal ion concentration (1 mM), Na^+^, Ni^2+^, Co^2+^, K^+^, and Ca^2+^ have an activating effect on TcPanD-R98H/K305S, while Fe^2+^, Cu^2+^, Zn^2+^, and Ba^2+^ have an obvious inhibiting effect, as shown in Figure 6. A high concentration of Ni^2+^ and Na^+^ (5 mM) has an obvious activating effect, while Zn^2+^, Cu^2+^, Ba^2+^, and Fe^2+^ have an inhibiting effect.

The kinetic parameters of TcPanD and TcPanD-R98H/K305S were determined while using L-aspartate as the substrate. Table 2 shows the kinetic parameters of TcPanD and TcPanD-R98H/K305S. The *K*_m_ value of TcPanD-R98H/K305S was 1.23 mM, the *V*_max_ of the reaction was 7.05 U·mg^−1^, the *K*_cat_ value was 7.64 s^−1^, and the *K*_cat_/*K*_m_ value was 6.21 s^−1^·mM^−1^. The *K*_m_ value of TcPanD was 1.35 mM, the *V*_max_ of the reaction was 2.88 U·mg^−1^, the *K*_m_ and *K*_cat_/*K*_m_ values of TcPanD were 3.12 s^−1^ and 2.31 s^−1^·mM^−1^, respectively. The mutant had lower *K*_m_ values than the wild TcPanD, which suggested that the mutant TcPanD-R98H/K305S had higher substrate affinity than that of TcPanD. The high *V*_max_ and *K*_cat_ value of TcPanD-R98H/K305S showed its higher catalytic activity than the wild type of TcPanD.

### 2.3. β-Alanine Production at a 5-L Scale by the Whole-Cell Catalysis

**Figure 7:**
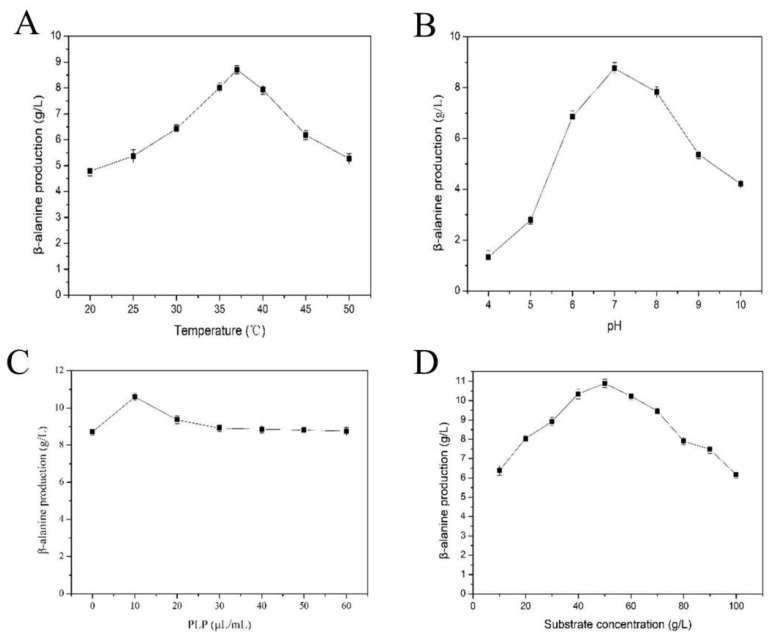
Optimization of the whole-cell biocatalysis conditions. (**A**), The effect of reaction temperature on β-alanine production; (**B**), The effect of reaction pH on β-alanine production; (**C**), The effect of PLP addition on β-alanine production; and, (**D**), The effect of substrate concentration on β-alanine production.

The biocatalytic conditions, including reaction temperature, pH, coenzyme PLP addition, and substrate concentration, were optimized using whole-cell catalysis to obtain a high level of β-alanine. The highest β-alanine production was obtained at the optimal condition (37 °C, pH 7.0, 10 μL/mL of PLP and 50 g/L of l-aspartate) for 10 h, as shown in Figure 7. Fed-Batch production of β-alanine at a 5-L scale was performed. Under the optimal condition, a total of 267 g/L l-aspartate was consumed, and 170.53 g/L β-alanine was obtained at 48 h with a molar conversion and production rate of 95.45% and 3.55 g·L^−1^·h^−1^. This result has been the highest yield of β-alanine by catalysis so far, and the mutant TcPanD-R98H/K305S avoids the inherent inactivity of the pyruvyl-dependent PanD and it has great potential for industrial purposes.

## 3. Materials and Methods

### 3.1. Materials

Restriction enzymes, plasmid extraction kit, and fragment purification kit were purchased from TAKARA (Dalian, China); Controlled Error-prone PCR Kit was purchased from TIANDZ (Beijing, China); ClonExpress II One Step Cloning Kit was purchased from Vazyme (Nanjing, China); yeast powder, peptone, Kanamycin, Ampicillin, Isopropyl-β-d-thiogalactoside, and l-aspartic acid were purchased from Sangon (Shanghai, China); 1,2-Diacetylbenzene was from Sigma-Aldrich (St. Louis, MO, USA). All of the chemicals used were of analytical grade.

### 3.2. Gene Cloning

The *TcPanD* gene (NCBI accession number of NP_001096055.1) was amplified from the genome of *Tribolium castaneum* and then cloned into the expression plasmid pET-28a (+). The recombinant plasmid pET28a-*TcPanD* was transformed into the competent *E. coli* BL21(DE3) cells. DNA sequencing confirmed the recombinant strains *E. coli* BL21/pET28a- *Tc*PanD.

### 3.3. Random Mutagenesis and Library Screening

The *TcPanD* gene was randomly mutated by error-prone PCR while using Controlled Error-Prone PCR Kit, and the mutation products were cloned into *Nco* Ι/*Xho* Ι sites of the expression plasmid pET-28a (+) (the primer sequences are shown in Table 3). These mutants were then transformed into the *E. coli* BL21(DE3) cells. The transformants were inoculated into LB medium containing 100 μg/mL kanamycin in 96-deep-well plates and cultured at 37 °C for 24 h. The cultures were inoculated with 50 μL of inoculum per well and then inoculated into a new 96-deep-well plate containing 500 μL of LB medium with 100 μg/mL kanamycin and 0.2 mM IPTG each well for induction. After being incubated at 30 °C for 24 h, the culture sample was centrifuged and 150 μL of cell lysate (50 mM of PBS buffer at pH 7.4, 1 mg/mL lysozyme) was added to each well. The cells were then repeatedly frozen and thawed at −80 °C and 37 °C three times, and the resulting solution was then centrifuged at 4000×*g* for 10 min. and the supernatant was used as the crude enzyme. A volume of 500 μL l-aspartate solution (50 g/L and adjusted to pH 7.0 with NaOH) was mixed with the crude enzyme and incubated at 37 °C for 30 min.

A high-throughput fluorometric method was used to screen the mutant with high TcPanD activity [19]. The screening system in 96-well plate was composed of 155 μL of solution (0.2 M sodium borate buffer, pH 9.5), 2.5 μL of 10 mg/mL o-diacetylbenzene in methanol, 2.5 μL of 5.7 mg/mL β-mercaptoethanol in ethanol, and 2.5 μL of the reaction mixture described above. The 96-well plate was quickly placed in the Infinite M200 spectrophotometer system (TECAN, Männedorf, Switzerland) and then shaken for 10 s to ensure the solution was completely mixed. The fluorescence with excitation and emission wavelength 355 and 445 nm in a 96-well plate was scanned at every 4 min. for two h. The highest value of the fluorescence that was observed in 2 h corresponded to the concentration of β-alanine.

### 3.4. Site-Directed Saturation Mutation

The overlap-extension PCR using primers, as shown in Table 4, constructed the site-directed saturation mutations of the *TcPanD* gene at the Arg98, Lys351, and Ile451 sites. The mutated genes were then cloned into the pET-28a (+) and transformed into the *E. coli* BL21(DE3) cells to construct the site-directed saturated mutation libraries and the high-throughput screening method described above was used to screen the mutants with high TcPanD activity.

### 3.5. Homology Modeling and Molecular Docking

The three-dimensional structural information and homology modeling of the TcPanD protein was predicted by its amino acid sequence using the online service MPI Bioinformatics Toolkit (https://toolkit.tuebingen.mpg.de/). The crystal structure of PanD from the human-derived cysteine sulfinic acid decarboxylase (PDB number: 2jis) was used as a template with a similarity of 60%. The structural model of TcPanD was obtained by the homology modeling strategy. The molecular docking simulation was performed by AutoDock Vina (Ver.1.1.2, Scripps Research Institute, La Jolla, CA, USA). The modeled protein structure was viewed using PyMol software (Ver. 2.7, Schrödinger, New York, NY, USA) and different zones were distinguished by different colours for analysis.

### 3.6. Protein Expression and Purification

The recombinant TcPanD was induced by adding 0.2 mM IPTG when the OD_600_ value of the culture reached 0.6–0.8. The induced cells were incubated at 30 °C for 12 h and then centrifuged at 12,000× *g* and 4 °C for 10 min. The cells were washed twice and then resuspended in 50 mM Tris-HCl buffer (pH 8.0), and then disrupted by the ultrasonic system to release the recombinant enzyme. Ni^2+^-affinity chromatography purified the crude enzyme on the ÄKTA purifier system (GE Healthcare, Pittsburgh, PA, USA). The active fractions were collected and stored at −20 °C for further analysis. The purity of the recombinant enzyme was detected by the 12% SDS-PAGE and the protein concentration was determined by the Bradford method assay while using bovine serum albumin (BSA) as the standard.

### 3.7. TcPanD Activity Assay

The TcPanD enzymatic activity was assayed in a reaction mixture containing 30 μL of the purified enzyme, 920 μL of 0.1 M phosphate buffer (pH 7.0) and 50 μL of 50 mM l-aspartate solution (adjusted to pH 7.0 with NaOH). The reaction was incubated at 37 °C for 20 min. and then stopped by adding 100 μL of 2 M NaOH. The samples were derivatized by 2,4-dinitrofluorobenzene and then analyzed by HPLC (Waters, Milford, MA, USA) under 360 nm absorbance. One unit of TcPanD activity is defined as the amount of enzyme that produces 1 μmol β-alanine in one minute under the described conditions.

### 3.8. The Effect of Coenzyme on the TcPanD Activity

The effects of different concentrations of pyridoxal 5’-phosphate (PLP, 0.1–0.8%) on the purified enzyme were investigated to obtain the optimal coenzyme addition for enzyme activity. The highest enzyme activity of the purified TcPanD enzyme was set to 100%.

### 3.9. Temperature Related to Optimum and Stability

Effects of different temperatures (20–90 °C) on the purified TcPanD were investigated to obtain the optimal temperature for enzyme activity. The purified enzyme was incubated at a temperature range of 4–60 °C for 0 h and 2 h, and the residual activity for each condition was respectively measured to investigate the thermal stability. The highest enzyme activity of the purified TcPanD was set to 100%.

### 3.10. pH Related to Optimum and Stability 

The effects of different pH (4.0–11.0) on the purified TcPanD were investigated to obtain the optimal pH for enzyme activity. The purified protein was incubated at different pH for 0 h and 2 h, and the residual activity for each condition was measured, respectively, to investigate the pH stability. The highest enzyme activity was set to 100%.

### 3.11. Effects of Different Metal Ions on the TcPanD Activity

The purified TcPanD was mixed with 1 mM and 5 mM metal ions (Co^2+^, Ca^2+^, K^+^, Zn^2+^, Cu^2+^, Ni^2+^, Fe^2+^, Fe^3+^, Ba^2+^, Na^+^, Mn^2+^, and Al^3+^), respectively, for 30 min. and the residual activities were measured under the above conditions. The enzyme activity without metal ions treatment was set to control and was taken as 100%.

### 3.12. Determination of the Kinetic Parameters

The TcPanD was determined while using the assay method described above, with the l-aspartate range from 0.5 to 100 mM as substrate. The reaction was sampled at 0, 1, 2, 4, 8, 12, 16, and 20 min. The initial reaction rate was determined based on the amount of β-alanine under different substrate concentrations. The Michaelis–Menten nonlinear fitting of the initial velocity and substrate concentration was used to determine the apparent kinetic parameters *K*_m_ value, *V*_max_ value, and turnover numbers *K*_cat_, respectively.

### 3.13. β-Alanine Production at a 5-L Scale

When considering the deviation of each batch of enzyme production and experimental operation error, the vacuum freeze-dried cells of *E.coli* carrying the mutant *Tc*PanD-R98H/K305S was used as the biocatalyst for β-alanine production. All of the optimizations were performed at a 50 mL flask level with 10 mL substrate containing 5 mL 200 mM sodium phosphate buffer (pH 7.0) and 5 mL of 100 g/L L-aspartate. The reaction was performed at 37 °C and stirred at 600 rpm by a magnetic stirrer RCT B S25 (IKA, Staufen, Germany). The effects of temperature (20–50 °C), buffer pH (4.0–10.0), coenzyme PLP addition (0–60 μL/mL), and substrate concentration (10–100 g/L) were investigated to optimize the conversion efficiency of l-aspartate to β-alanine to optimize the conversion efficiency of whole-cell catalysis. Three sets of parallel experiments were performed for each factor, and the results were averaged.

The *E. coli* BL21(DE3) carrying the mutant gene *TcPanD-R98H/K305S* was cultured in a 5 L bioreactor, and the cells were collected and then transferred into a 1 L bioconversion mixture (OD_600_ = 100) containing 50 g/L l-aspartate. The whole-cell bioconversion was performed at 37 °C and 600 rpm. The l-aspartate was fed to 50 g/L when its concentration dropped to 10 g/L during the whole bioconversion process.

## 4. Conclusions

The PLP-dependent PanD avoids the inherent inactivity of the pyruvyl-dependent PanD during a long-term reaction in industrial production; thus, it is an ideal catalyst for β-alanine production. The random mutation coupling with the site-directed mutation is an effective method to engineer the TcPanD, improving its activity and stability and making it more suitable for industrial production. The scale-up production of β-alanine using the TcPanD mutant validates the availability of this enzyme mutant when applied in industrial production.

## Figures and Tables

**Table 1:** Purification table of *Tc*PanD and *Tc*PanD-R98H/K305S.

	Purification Steps	Total Protein (mg)	Total Activity (U)	Specific Activity (U/mg)	Fold Purification	Yield (%)
*Tc*PanD	Crude enzyme	90.81	122.05	1.34	1	100
	Ni^+^ column affinity chromatography	28.97	71.09	2.45	1.83	58.25
	Ultrafiltration concentration	19.05	54.78	2.88	2.15	44.88
*Tc*PanD-R98H/K305S	Crude enzyme	120.55	306.19	2.54	1	100
	Ni^+^ column affinity chromatography	33.72	164.14	4.87	1.92	53.61
	Ultrafiltration concentration	18.15	127.90	7.05	2.78	41.77

**Table 2:** Kinetic parameters of *Tc*PanD and *Tc*PanD-R98H/K305S.

	*K*_m_ (mM)	*V*_max_ (U mg^−1^)	*K*_cat_ (s^−1^)	*K*_cat_/*K*_m_ (s^−1^· mM^−1^)
*Tc*PanD	1.35	2.88	3.12	2.31
*Tc*PanD-R98H/K305S	1.23	7.05	7.64	6.21

**Table 3:** Primer sequence for Error-Prone PCR.

	Primer Sequence	Restriction Endonuclease	Recognition Sequence
Forward primer	5’- TAAGAAGGAGATATACCATGGGCATGCCCGCTACCG-3’	*Nco* Ι	CCATGG
Reverse primer	5’- GTGGTGGTGGTGGTGCTCGAGTAAGTCCGAGCCAAGACG-3’	*Xho* Ι	CTCGAG

**Table 4:** Primer sequence for site-directed saturation mutation.

	Primer Sequence	Altered Bases
98-For	5’-GAACCGGAGGAACTTNNNCGCTTGATGGATTTT-3’	NNN
98-Rev	5’-AAAATCCATCAAGCGNNNAAGTTCCTCCGGTTC-3’	
305-For	5’-TTCGATCCCATTGAGNNNATCGCCGACGTGTGT-3’	
305-Rev	5’-ACACACGTCGGCGATNNNCTCAATGGGATCGAA-3’	
451-For	5’-GGTTTCGAAATGGTANNNGCCGAGCCCGAATAT-3’	
451-Rev	5’-ATATTCGGGCTCGGCNNNTACCATTTCGAAACC-3’	

Underline: the site-mutation amino acid point.
